# Paradoxical ventilator associated pneumonia incidences among selective digestive decontamination studies versus other studies of mechanically ventilated patients: benchmarking the evidence base

**DOI:** 10.1186/cc9406

**Published:** 2011-01-07

**Authors:** James C Hurley

**Affiliations:** 1 Rural Health Academic Centre, Melbourne Medical School, The University of Melbourne, 'Dunvegan' 806 Mair St., Ballarat, Victoria 3350, Australia; 2 Infection Control Committees, Ballarat Health Services and St John of God Hospital, Ballarat, and Physician, Division of Internal Medicine, Ballarat Health Services, 101 Drummond St., N, Ballarat, 3350, Victoria, Australia

## Abstract

**Introduction:**

Selective digestive decontamination (SDD) appears to have a more compelling evidence base than non-antimicrobial methods for the prevention of ventilator associated pneumonia (VAP). However, the striking variability in ventilator associated pneumonia-incidence proportion (VAP-IP) among the SDD studies remains unexplained and a postulated contextual effect remains untested for.

**Methods:**

Nine reviews were used to source 45 observational (benchmark) groups and 137 component (control and intervention) groups of studies of SDD and studies of three non-antimicrobial methods of VAP prevention. The logit VAP-IP data were summarized by meta-analysis using random effects methods and the associated heterogeneity (tau^2^) was measured. As group level predictors of logit VAP-IP, the mode of VAP diagnosis, proportion of trauma admissions, the proportion receiving prolonged ventilation and the intervention method under study were examined in meta-regression models containing the benchmark groups together with either the control (models 1 to 3) or intervention (models 4 to 6) groups of the prevention studies.

**Results:**

The VAP-IP benchmark derived here is 22.1% (95% confidence interval; 95% CI; 19.2 to 25.5; tau^2 ^0.34) whereas the mean VAP-IP of control groups from studies of SDD and of non-antimicrobial methods, is 35.7 (29.7 to 41.8; tau^2 ^0.63) versus 20.4 (17.2 to 24.0; tau^2 ^0.41), respectively (*P *< 0.001). The disparity between the benchmark groups and the control groups of the SDD studies, which was most apparent for the highest quality studies, could not be explained in the meta-regression models after adjusting for various group level factors. The mean VAP-IP (95% CI) of intervention groups is 16.0 (12.6 to 20.3; tau^2 ^0.59) and 17.1 (14.2 to 20.3; tau^2 ^0.35) for SDD studies versus studies of non-antimicrobial methods, respectively.

**Conclusions:**

The VAP-IP among the intervention groups within the SDD evidence base is less variable and more similar to the benchmark than among the control groups. These paradoxical observations cannot readily be explained. The interpretation of the SDD evidence base cannot proceed without further consideration of this contextual effect.

## Introduction

Colonization and infection with bacteria occurs commonly in patients receiving mechanical ventilation (MV) [1-5]. The use of selective digestive decontamination (SDD) is an approach to prevent colonization and pneumonia in this patient group [6]. Systematic reviews of more than 30 controlled studies of SDD provide compelling evidence of reductions in VAP of >50% [6] versus marginally significant reductions of <20% with non-antibiotic methods of prevention such as those based on the management of gastric pH [7], tracheal suction [8], or humidification [9].

That SDD could create a contextual effect in the intensive care unit through cross colonization between patients of concurrent control and study groups was postulated in the original 1984 study [10] and others [11], which were intentionally non-concurrent in design. This postulate remains untested. Moreover, the VAP-IP of control groups of SDD studies is highly variable, particularly among SDD studies with a concurrent design [12]. To account for this variability and to test the original postulate would require an external benchmark of VAP-IP.

Four recent factors enable a benchmarking of the VAP-IP among the component groups of the SDD evidence base. First, five reviews [1-5] have independently estimated the expected VAP-IP range for observational groups and enable the derivation of a benchmark. Second, the key studies in the evidence base for SDD and for comparison, three non-antibiotic methods of VAP prevention, are identified in four large systematic reviews [6-9]. Third, various group level factors, which may be explanatory toward the VAP incidence, are identified in all of the studies. Finally, heterogeneity among study results can now be measured and incorporated in the derivation of a prediction range using recently developed random effects methods of meta-analysis and displayed using a caterpillar plot [13,14].

## Materials and methods

### Overview

There are four objectives here: First, to derive a VAP-IP benchmark and prediction range derived from observational (benchmark) groups. Second, to summarize VAP-IP separately for the control and intervention groups from studies of two broad approaches to VAP prevention that have been included in systematic reviews; studies of SDD versus studies of non-anti-microbial methods of VAP prevention. Third, to assess the dispersion among the group specific VAP-IP of control groups and intervention groups versus the VAP-IP benchmark using caterpillar plots. Finally, to assess the impact of group level factors as possible explanatory variables toward the group specific VAP-IP in meta-regression models that include both the benchmark and the prevention study groups.

### Study selection and component group designations

This analysis is limited to component groups from studies of patients receiving mechanical ventilation as abstracted in nine published reviews (four non-systematic and five systematic) of VAP incidence and specific VAP prevention methods [1-9]. The unit of analysis here is the component patient group, whether observational (benchmark) [1-5], or control or intervention groups from studies of various methods of VAP prevention [6-9].

The inclusion criterion for this analysis was a study of adult patients receiving prolonged mechanical ventilation in intensive care units (ICUs) for which VAP-IP and denominator data had been abstracted in one of the nine reviews [1-9]. The exclusion criteria as specified in the Cochrane review [6] are applied to achieve harmonization across the studies obtained from all nine reviews. That is; studies based on specific pre-selected types of patients (patients undergoing elective esophageal resection, cardiac or gastric surgery, liver transplant or suffering from acute liver failure), studies of non-ICU populations, populations for which the proportion receiving MV for >24 hours was <50% and studies for which VAP-IP data were not available. Also, studies of pediatric populations, and studies published before 1984 do not appear among the studies abstracted in the review of Liberati *et al. *[6] and these study types are also excluded.

### Categories of benchmark and component groups

The benchmark groups are those groups of observational studies as abstracted in one of five reviews of VAP incidence [1-5]. Any intervention study abstracted in one of these five reviews of VAP-IP incidence was not used in the derivation of the benchmark.

The component groups of studies of non-antimicrobial methods of VAP prevention are as abstracted in one of three systematic reviews of various methods of gastric acid suppression [7], open versus closed methods of tracheal suction [8], or passive versus active humidification [9] as methods of VAP prevention. In the gastric acid studies, the interventions studied were those that might suppress gastric acid (for example, ranitidine or antacid treatment) versus interventions that did not (for example, no treatment or sucralfate) [7]. The designation of control and intervention groups were as indicated in the systematic reviews of open (control) versus closed (intervention) methods of tracheal suction [8] and passive (HH, control) versus active (HME, intervention) humidification [9]. The component groups from the studies of SDD are as abstracted in the Cochrane review [6].

### Data extraction

The primary outcome is the VAP-IP, which is the incidence of ventilator associated pneumonia per 100 patients. The VAP-IP and its denominator were taken for all component groups as abstracted in the review documents in which they appeared.

Additional information abstracted directly from the original publication was whether the mode of VAP diagnosis required bronchoscopic sampling versus tracheal sampling methods, whether <90% of patients received at least 24 hours of mechanical ventilation, and the proportion of patients admitted to the ICU for trauma. The scoring of study quality was also abstracted from each systematic review. However, each systematic review used different quality scoring systems and scoring was not used in the non-systematic reviews. The indicator of highest study quality in this analysis was whether the study received a majority score in the source systematic review. Data were extrapolated from tables and figures if not available in the text. Care was taken to stratify patient groups appearing across more than one publication.

### Caterpillar plots

A caterpillar plot is a forest plot-like display of group specific odds and 95% confidence intervals with the studies listed in rank order of increasing event rate. This display reveals both the overall symmetry of the individual group results and their deviation from the overall mean. This display shows the impact of group size with the larger groups, having greater precision, expected to deviate less from the summary or benchmark.

### Statistical methods

The VAP-IP data were converted to logits for analysis as follows; if D represents the denominator, N represents the numerator, and R represents the proportion (N/D) of the VAP-IP, the logit(VAP-IP) is log(N/(D-N)) and its variance is 1/(D*R*(1-R)) [15,16]. This variance formula was used to calculate the group specific 95% confidence intervals. Using these calculated logits and logit variances, the metan command [17] in STATA (release 11.0, STATA Corp., College Station, TX, USA) generates summary logits by a random effects method together with the standard errors (SE) and tau^2^, which are measures of within and between group variances, respectively, and the associated 95% CI's. The metan command also generates the caterpillar plots of the group specific logits and 95% CI's.

The VAP-IP benchmark was derived as the mean logit VAP-IP and 95% confidence interval derived together with a 95% prediction interval. The later is calculated using the metan command as mean ± 1.96 * (SE^2 ^+ tau^2^)^0.5 ^[17]. In each of the caterpillar plots, both the overall VAP-IP mean derived from the groups in the plot and the 95% prediction interval derived from VAP-IP benchmark range are displayed.

To test the stability of the benchmark, five replicate derivations of the VAP-IP benchmark were derived using the VAP-IP data abstracted from the four non-systematic and one systematic reviews individually [1-5].

### Meta-regression

The calculated logits and logit variances were used with the metareg command [18] in STATA (release 11.0, STATA Corp.) to perform meta-regression models that incorporate group level factors as predictors. There are six meta-regression models of logit VAP-IP including the benchmark groups with either the control (models 1 to 3) or the intervention (models 4 to 6) groups of the prevention studies. Models 1 and 4 include group membership (benchmark, SDD study or non-antimicrobial method study), as the only predictors. Models 2 and 5 include three additional group level properties as predictor variables; whether <90% of patients in the group received >24 hours of MV, whether the mode of diagnosis of VAP required bronchoscopic sampling and the proportion of trauma admissions to the ICU. Models 3 and 6 replicate models 2 and 5 but are limited to those studies that had received majority quality scores in the source systematic reviews. Regression coefficients were compared using the lincom (linear combination) post-estimation command in STATA.

### Sensitivity analysis

Meta-regressions models 2 and 4 were repeated after exclusion of studies for which the proportion of patients receiving >24 hours of mechanical ventilation was <90% or unknown. Also, meta-regressions models 3 and 6 were repeated with component groups from 19 studies of SDD that had received a quality score of one out of two included.

## Results

There were 45 observational benchmark groups (Additional file 1) [19-63] and 137 component groups (Additional files 2 and 3) [64-131] derived from nine reviews [1-9]. The characteristics of the studies and the groups are summarized in Table 1. Most studies had been published in the 1990's. Compared to the benchmark groups, the component groups of the studies of VAP prevention methods differed in the following respects; they had fewer patients per group (*P *= 0.001), fewer had bronchoscopic sampling performed for VAP diagnosis (*P *= 0.003) and admissions for trauma among them were more frequent (*P *= 0.01). The studies of non-antimicrobial methods more often attained majority quality scores than did studies of SDD in the respective systematic reviews (*P *= 0.006).

**Table 1:** Characteristics of studies and component groups

	Studies and component groups		
	
	Observational (Benchmark)	Non-antimicrobial	SDD
Studies			
Originating review [ref]	[1 to 5]^a^	[7 to 9] ^b^	[6]^c^
Number of studies ^d, e^	45	35	33
Bronchoscopic sampling ^f^	23	5	8
Publication year (IQR) ^g^	1990 to 2000	1994 to 2000	1991 to 1997
European ^h, i^	28	19	30
Majority quality score ^j, k^	NA	16	4
MV for > 24 hours for <90% ^l^	5	2	4
Component groups			
Numbers of patients per group; median (IQR) ^m, n^	264; 83 to 567	54; 29 to 92	57; 33 to 130
Days of ventilation; median (IQR)^o^	10.8; 8.0 to 12.8	8.9; 6.7 to 13.4	10.5; 9.0 to 15.0
% trauma patients; median (IQR)^p^	12; 2 to 35	15; 10 to 59	34; 18 to 78
VAP - IP; median IQR (n)			
Observational (benchmark)	22.0; 15 to 30.8 (45)	NA	NA
Control		17.5; 12.5 to 28.9 (35)	42; 21.6 to 51(33)
Intervention		15.4; 9.1 to 22.7 (35)	13.3; 7.1 to 24.4 (34)

n, number; NA, not available; IQR, inter-quartile range; SDD, Selective Digestive Decontamination; VAP-IP, ventilator associated pneumonia incidence proportion.

^a ^These data were sourced as follows; George, 1993 [1] (Table 1), Cook and Kollef, 1998 [2] (Table 1), Chastre and Fagon, [3] 2002 (Table 1), Bergmans and Bonten, [4] 2004 (Table 22.5), Safdar *et al.*, [5] 2005 (Table 1).

^b ^The following systematic reviews were the source for these studies; Messori *et al.*, [7] 2000 (Tables 5-7), Subirana *et al.*, [8] 2007 (Table 7), and Siempos *et al.*, [9] 2007 (Table 2) were the sources for these studies.

^c ^Liberati *et al.*, [6] 2009 (Analysis 1.5, and 2.5) was the source for these studies.

^d ^Reasons for benchmark group exclusions; 9 studies of defined patient populations (pediatric, cardio-thoracic surgery, liver transplantation, ARDS), 12 studies with <50% of patients receiving MV >24 hours, 6 studies published prior to 1983, or 6 intervention studies.

^e ^Reasons for VAP prevention study exclusions; 2 studies of defined patient populations (cardio-thoracic surgery, liver transplantation), or VAP-IP data not available (12 studies).

^f ^Comparison of mode of diagnosis, chisquared test = 13.5, two degrees of freedom *P *= 0.001.

^g ^Data is inter-quartile range (IQR).

^h ^Originating from a member state of the European Union as at 2010 or Switzerland or Norway.

^i ^Comparison of European origin, benchmark versus prevention studies, chisquared test = 1.4, one degree of freedom *P *= 0.24.

^j ^A majority quality score as assessed in the originating systematic reviews which had been scored out of a possible 10 [7], 4 [8], 5 [9] and 2 [6] criteria.

^k ^Comparison of high quality score, chisquared test = 7.43, one degree of freedom *P *= 0.006.

^l ^Number of studies for which the proportion of patients ventilated for >24 hours was <90% or not stated.

^m ^Data is median and inter-quartile range (IQR).

^n ^Comparison of group sizes, chisquared test = 34.7, two degrees of freedom *P *= 0.0001.

^o ^Comparison of days of ventilation, chisquared test = 1.4, two degrees of freedom *P *= 0.49.

^p ^Comparison of percent of trauma patients, chisquared test = 7.5, two degrees of freedom *P *= 0.02.

**Figure 1:**
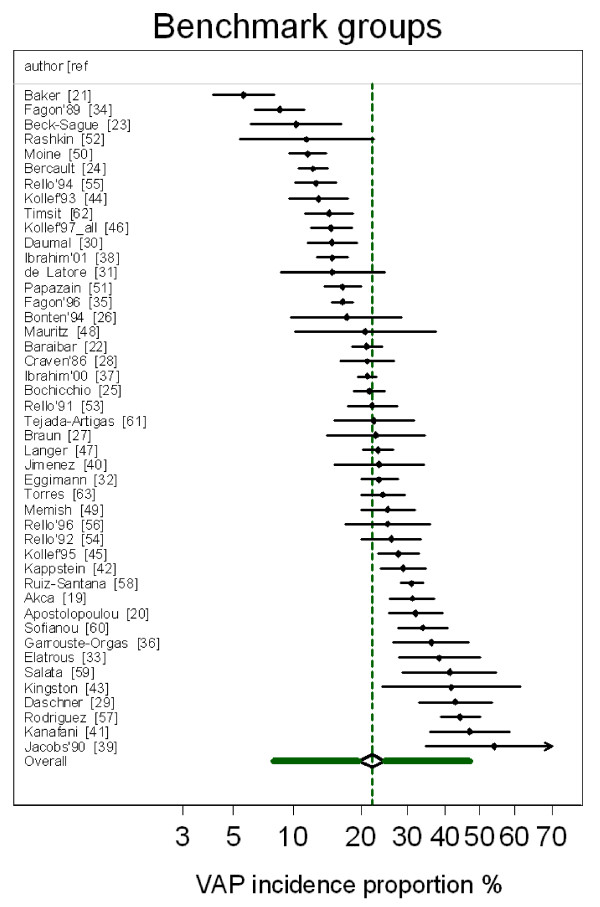
**Caterpillar plot: observational (benchmark) groups and derived benchmark**. Caterpillar plot of the group specific (small diamonds) VAP-IP and 95% CI of observational benchmark groups together with the summary VAP-IP (dotted green vertical line), 95% CI (large open diamond) and 95% prediction interval (solid green horizontal line). Note that the x axis is a logit scale. The VAP-IP data is as abstracted in four non-systematic and one systematic review [1-5].

The VAP-IP benchmark derived from all 45 observational (benchmark) groups is 22.1% with a 95% confidence interval of 19.0% to 25.5%, and with a 95% prediction interval of 8.6% to 47.3% (Figure 1). The five replicate estimates of the benchmark using the abstracted VAP-IP data from the observational (benchmark) groups abstracted in each of the four non-systematic and one systematic reviews were each within five percentage points of the benchmark derived using the abstracted VAP-IP data from all 45 observational (benchmark) groups (Table 2). Among the benchmark groups, there was no significant trend in VAP-IP versus publication year (data not shown, *P *= 0.47). A summary VAP-IP derived from benchmark groups originating from European centres and non-European centres were each within two percentage points of the benchmark (Table 2).

**Table 2:** Sources and replicate estimates of VAP-IP benchmark range

	VAP-IP range estimates (%)			
	
Source review, Year	Original ^a^		Re-analysis ^b^	
		
		N	Mean; 95% CI	N ^c^
George, 1993 [1]^d^	8 to 54	23	23.7; 18.1 to 30.4	11
Cook and Kollef, 1998 [2]^d^	13 to 38	8	21.4; 17.5 to 25.7	8
Chastre and Fagon, 2002 [3]^d^	8 to 28	10	17.2; 13.4 to 22.1	10
Bergmans and Bonten, 2004 [4]^d^	8.6 to 65	15	20.6; 16.1 to 26.1	14
Safdar, *et al.*, 2005 [5]^e^	7 to 12.5	28	21.1; 17.9 to 24.4	25
All five reviews [1-5]			22.1; 19.2 to 25.5 ^f^	45
				
European benchmark groups [1-5]			21.2; 18.1 to 24.6	28
Non- European benchmark groups [1-5]			23.9; 19.6 to 28.8	17

VAP-IP, Ventilator associated pneumonia incidence proportion, N, number of groups.

^a. ^The original VAP-IP range and numbers of abstracted studies (N) had been derived in the source systematic review by the following methods; minimum-maximum study VAP-IP values [1-3] or mean VAP-IP weighted by study size [5] or unstated [4].

b. Re-analysis VAP-IP range derived by meta-analysis using the abstracted VAP-IP data and numbers of eligible abstracted studies (N) from each systematic review.

c. The number of eligible groups (N) from each systematic review included in the re-analysis. Note, the column does not tally as some studies were abstracted in more than one systematic review.

d. Non-systematic review.

e. Systematic review.

f. This is the benchmark range.

**Figure 2:**
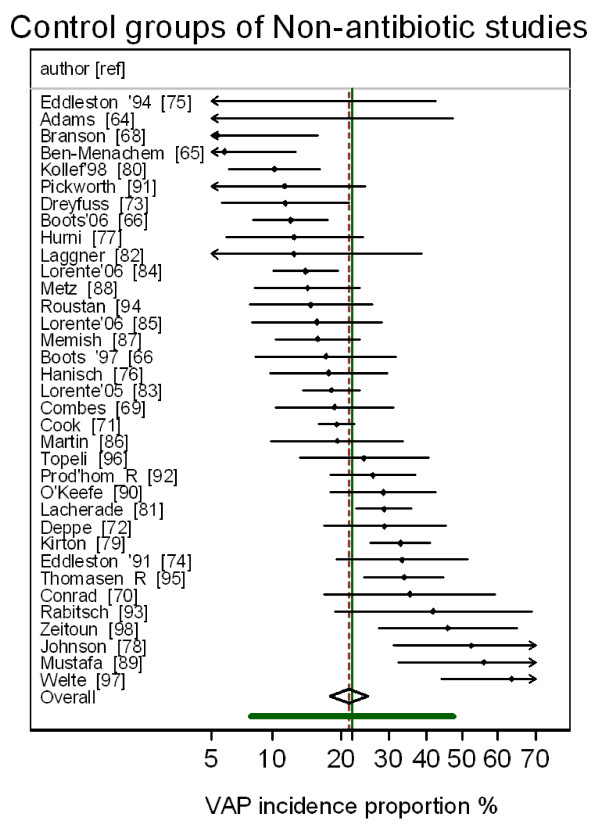
**Caterpillar plot: control groups of studies of non-antimicrobial methods of VAP prevention**. Caterpillar plot of the group specific (small diamonds) and summary (broken vertical line) VAP-IP and 95% CI (large open diamond) of control groups of studies of non-antimicrobial methods of VAP prevention. The VAP-IP data is as abstracted in three systematic reviews [7-9]. For comparison, the VAP-IP benchmark (solid green vertical line) and prediction interval (solid green horizontal line) derived from the benchmark groups from Figure 1 is also shown. Note that the x axis is a logit scale.

**Figure 3:**
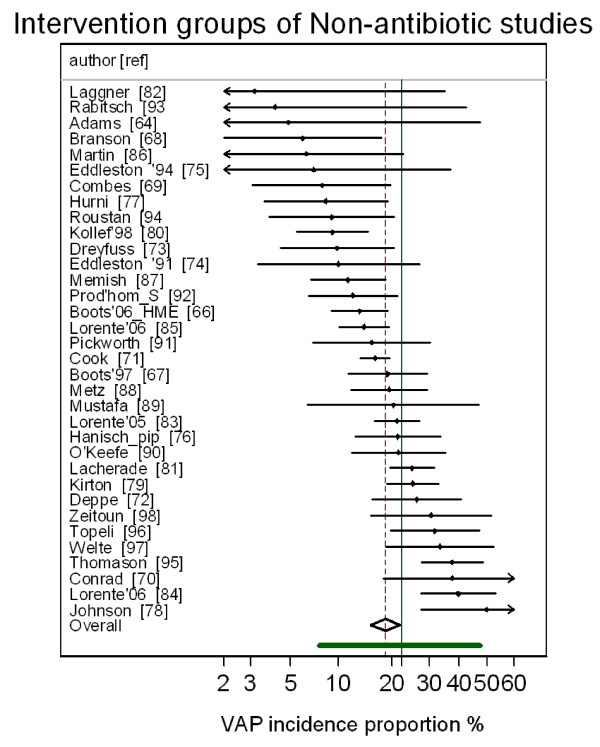
**Caterpillar plot: intervention groups of studies of non-antimicrobial methods of VAP prevention**. Caterpillar plot of the group specific (small diamonds) and summary (broken vertical line) VAP-IP and 95% CI (large open diamond) of intervention groups of studies of non-antimicrobial methods of VAP prevention. The VAP-IP data is as abstracted in three systematic reviews [7-9]. For comparison, the VAP-IP benchmark (solid green vertical line) and prediction interval (solid green horizontal line) derived from the benchmark groups from Figure 1 is also shown. Note that the x axis is a logit scale.

**Figure 4:**
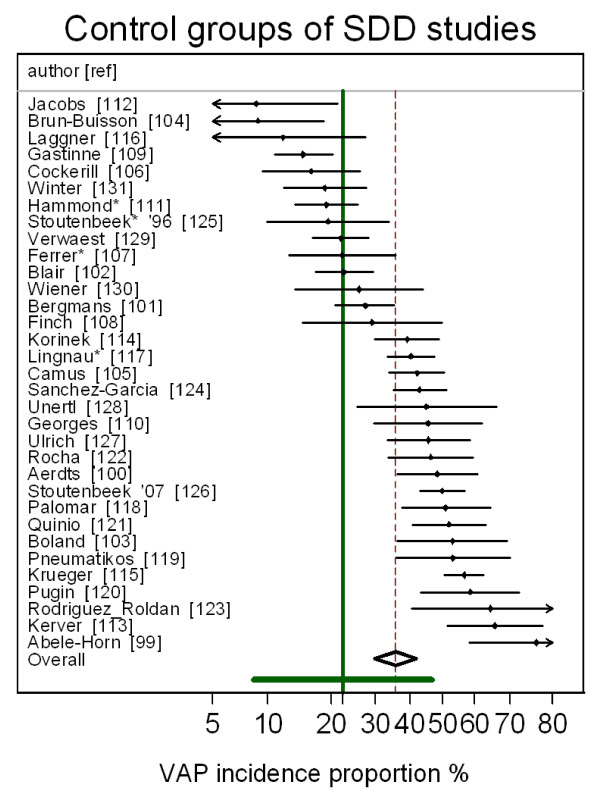
**Caterpillar plot: control groups of SDD studies**. Caterpillar plot of the group specific (small diamonds) and summary (broken vertical line) VAP-IP and 95% CI (large open diamond) of control groups of SDD studies. Four control groups from duplex studies that is, all control group patients routinely received systemic antibiotics, are indicated by an asterix next to the author name and NC indicates non-concurrent. The VAP-IP data is as abstracted in Liberati *et al. *[6]. For comparison, the VAP-IP benchmark (solid green vertical line) and prediction interval (solid green horizontal line) derived from the benchmark groups from Figure 1 is also shown. Note that the x axis is a logit scale.

**Figure 5:**
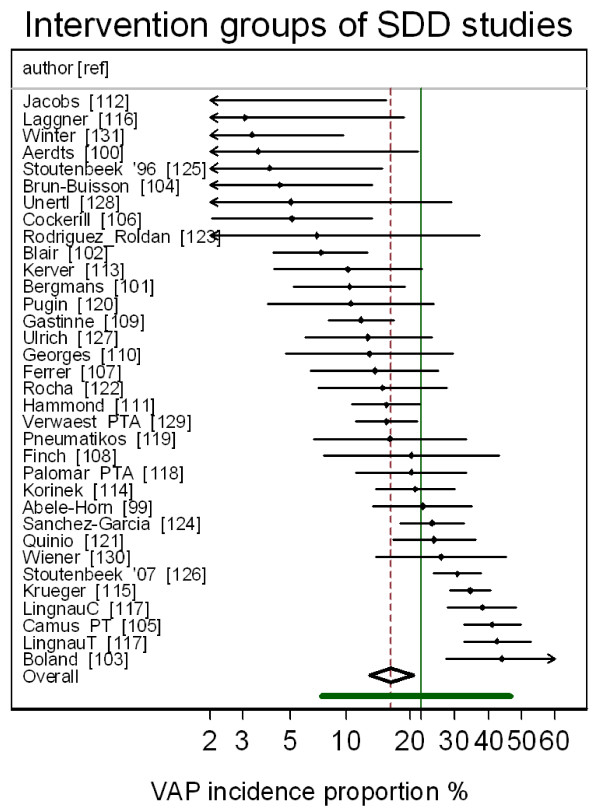
**Caterpillar plot: intervention groups of SDD studies**. Caterpillar plot of the group specific (small diamonds) and summary (broken vertical line) VAP-IP and 95% CI (large open diamond) of intervention groups of SDD studies. The VAP-IP data is as abstracted in Liberati *et al.*[6]. For comparison, the VAP-IP benchmark (solid green vertical line) and prediction interval (solid green horizontal line) derived from the benchmark groups from Figure 1 is also shown. Note that the x axis is a logit scale.

The group specific and summary VAP-IP's for the component groups of the prevention studies are displayed in Figures 2, 3, 4, 5 and the summary VAP-IPs are tabulated in Table 3. The I^2 ^associated with the summary estimates ranged between 74% and 93%. The distribution of the group specific VAP-IPs of the control groups of the SDD studies differs in five ways versus the distribution of the group specific VAP-IPs among the control groups of the studies of non-antibiotic methods; the mean and tau^2 ^are 50% higher (Table 3) and the interquartile range (IQR) (Table 1) and confidence intervals (Table 3) are both 50% wider. Moreover, the median VAP-IP (Table 1) of the control groups of the SDD studies is more than five percentage points higher than the mean (Table 3), a finding which indicates a positive skew.

**Table 3:** Study quality and summary estimates of VAP-IP

Strata of groups	All studies				Highest quality studies ^a^			
		
	Mean; 95% CI	N	SE	tau^2^	Mean; 95% CI	N	SE	tau^2^
Observational (benchmark) groups	22.1; 19.2 to 25.5	45	0.09	0.34	22.1; 19.2 to 25.5	45	0.09	0.34
Studies of VAP prevention using non-antimicrobial methods								
Control groups	20.4; 17.2 to 24.0	35	0.13	0.41	18.4; 14.9 to 22.3	16	0.16	0.24
Intervention groups	17.1; 14.2 to 20.3	35	0.13	0.35	15.3; 12.6 to 18.7	16	0.13	0.16
Studies of methods of VAP prevention using SDD								
Control groups	35.7; 29.7 to 41.8	33	0.15	0.63	44.7; 31.1 to 59.3 ^b^	4	0.39	0.52
Intervention groups	16.0; 12.6 to 20.3	34	0.16	0.59	18.5; 9.9 to 32.1 ^c^	4	0.53	0.90

CI, confidence interval; N, number; SE, Standard error; SDD, Selective Digestive Decontamination; VAP, Ventilator associated pneumonia.

a. The study quality scoring was as defined in the source systematic reviews. The highest quality score is a majority quality score as assessed in the originating systematic reviews which had been scored out of a possible 10 [7], 4 [8], 5 [9] and 2 [6] criteria. Benchmark groups had not been rated with a quality score in the review sources and for this analysis are considered to be of equal quality.

b. Summary estimates derived for 24 control groups of SDD studies which had a high quality score (1 out of 2) was 34.8; 28.7 to 58.5 (se = 0.15, tau^2 ^= 0.43).

c. Summary estimates derived for 25 intervention groups of SDD studies which had a high quality score (1 out of 2) was 17.9; 13.8 to 23.1 (se = 0.18, tau^2 ^= 0.60).

The differences in distributions of VAP-IP among the component groups of the prevention studies are also apparent in the caterpillar plots (Figures 2, 3, 4, 5) in that 11 of the 33 control groups of the SDD studies versus only 3 of the 35 control groups of the non-antibiotic studies have group specific VAP-IP's which are above the benchmark 95% prediction interval. Four of the control groups with VAP-IP within the benchmark prediction range were control groups from SDD studies that had a duplex design; that is, all control group patients routinely received systemic antibiotics.

The disparities in summary VAP-IP among the component groups of the prevention studies versus the benchmark remained apparent in analyses limited to the highest quality studies (Table 3). The mean VAP-IP of the control groups of highest quality SDD studies were 22 percentage points higher than the benchmark. By contrast, for all other component groups the summary VAP-IP's were within seven percentage points of the benchmark whether derived from the highest quality studies or all studies.

### Meta-regression models

Three meta-regression models were performed as described in the methods to evaluate several group level properties as predictors of the group specific logit VAP-IP's of the control (Table 4) and intervention (Table 5) groups versus the benchmark groups.

**Table 4:** Meta-regression models 1-3: benchmark and control groups

	Meta-regression analysis of logit VAP-IP		
	
Factor	Coefficient ^a^	95% confidence interval	*P*
Simple model (model 1; all studies)			
Benchmark groups (reference group)	-1.26	-1.47 to -1.05	<0.001
Non-antimicrobial series	-0.10	-0.44 to +0.24	0.56
SDD series	+0.67	+0.34 to +1.00	<0.001
Full model (model 2; all studies)			
Benchmark groups (reference group)	-1.07	-1.37 to -0.77	<0.001
Non-antimicrobial series	-0.38	-0.74 to -0.03	0.04
SDD series	+0.48	+0.16 to +0.81	0.004
Mode of diagnosis ^b^	-0.38	-0.68 to -0.08	0.01
Proportion trauma admissions ^c^	+0.30	-0.10 to +0.70	0.14
<90% ventilated patients ^d^	-0.41	-0.88 to +0.06	0.09
Full model (model 3; highest quality studies)			
Benchmark groups (reference group)	-0.98	-1.27 to -0.66	<0.001
Non-antimicrobial series	-0.35	-0.75 to +0.06	0.09
SDD series	+0.82	+0.14 to +1.50	0.019
Mode of diagnosis ^b^	-0.38	-0.72 to -0.04	0.03
Proportion trauma admissions ^c^	-0.13	-0.58 to +0.33	0.58
<90% ventilated patients ^d^	-0.31	-0.83 to +0.22	0.24

SDD, Selective Digestive Decontamination; VAP-IP, Ventilator associated pneumonia incidence proportion.

a. Interpretation. The benchmark groups in each model form the reference group and the size of this coefficient equals the difference in logits from 0 (a logit equal to 0 equates to a proportion of 50%; a logit equal to -1.26 equates to a proportion of 22.1%). The other coefficients in each model represent the additional difference in logits for groups positive for that factor versus the reference group.

b. For diagnosis using bronchoscopic versus tracheal based sampling.

c. Per 100% of admissions being for trauma.

d. For studies for which <90% of patients received >24 hours of mechanical ventilation.

**Table 5:** Meta-regression models 4-6: benchmark and intervention groups

	Meta-regression analysis of logit VAP-IP		
	
Factor	Coefficient ^a^	95% confidence interval	*P*
Simple model (model 4; all studies)			
Benchmark groups (reference group)	-1.26	-1.46 to -1.06	<0.001
Non-antimicrobial series	-0.32	-0.64 to -0.01	0.054
SDD series	-0.37	-0.70 to -0.03	0.03
Full model (model 5; all studies)			
Benchmark groups (reference group)	-1.19	-1.50 to -0.89	<0.001
Non-antimicrobial series	-0.46	-0.84 to -0.08	0.019
SDD series	-0.54	-0.88 to -0.18	0.003
Mode of diagnosis ^b^	-0.19	-0.50 to +0.13	0.24
Proportion trauma admissions ^c^	+0.34	-0.07 to +0.76	0.11
<90% ventilated patients ^d^	-0.30	-0.77 to +0.17	0.21
Full model (model 6; highest quality studies)			
Benchmark groups (reference group)	-1.05	-1.36 to -0.74	<0.001
Non-antimicrobial series	-0.57	-0.99 to -0.16	0.008
SDD series	-0.35	-1.09 to +0.39	0.34
Mode of diagnosis ^b^	-0.30	-0.64 to +0.04	0.08
Proportion trauma admissions ^c^	-0.06	-0.52 to +0.41	0.81
<90% ventilated patients ^d^	-0.11	-0.60 to +0.38	0.67

SDD, Selective Digestive Decontamination; VAP-IP, Ventilator associated pneumonia incidence proportion.

a. Interpretation. The benchmark groups in each model form the reference group and the size of this coefficient equals the difference in logits from 0 (a logit equal to 0 equates to a proportion of 50%; a logit equal to -1.26 equates to a proportion of 22.1%). The other coefficients in each model represent the additional difference in logits for groups positive for that factor versus the reference group.

b. For diagnosis using bronchoscopic versus tracheal based sampling

c. Per 100% of admissions being for trauma.

d. For studies for which <90% of patients received >24 hours of mechanical ventilation.

For the control groups versus the benchmark groups (Table 4; meta-regression models 1 to 3), membership of a control group of an SDD study was a consistently positive predictor. For the intervention groups versus the benchmark groups (Table 5; meta-regression models 4 to 6), membership of an intervention group of an SDD study was a negative predictor of logit VAP-IP but not consistently significant.

In comparing these factors in the meta-regression models, membership of a control group of an SDD study differed significantly versus membership of a control group of a non-antibiotic study in model 1 (*P *< 0.001), model 2 (*P *< 0.001) and model 3 (*P *= 0.003). By contrast, membership of an intervention group of an SDD study did not differ significantly versus membership of an intervention group of a non-antibiotic study as a predictor in model 4 (*P *= 0.7), model 5 (*P *= 0.6) or model 6 (*P *= 0.3).

Meta-regressions models 2 and 4 were repeated after exclusion of studies for which the proportion of patients receiving >24 hours of mechanical ventilation was <90% or unknown. Also, meta-regressions models 3 and 6 were repeated with component groups from 19 studies of SDD that had received a quality score of one out of two included. With both of these re-analyses, the findings were replicated (data not shown).

## Discussion

The present analysis has identified unexplained and paradoxical discrepancies among the VAP-IP of control groups and the intervention groups of SDD studies versus the benchmark and versus groups of other studies aggregated from reviews of other methods of VAP prevention. There were several analytic and statistical issues that needed to be addressed to execute this analysis.

The first analytic issue is the method of study selection. The objective here was to evaluate the evidence base as represented within systematic and other reviews. Hence a new literature search was not undertaken but the analysis was specifically limited to studies identified in nine published reviews and to the use of those studies exclusively. This narrowed focus allows scrutiny of the component groups that form an entire evidence base [6-9]. The three systematic reviews of non-antibiotic methods of VAP prevention were chosen because they were the largest available.

The second analytic issue is the method of abstracting VAP-IP data. The use of abstracted data from the reviews rather than from the published studies maintains objectivity and facilitates independent verification as all the data is readily identifiable in the reviews. Of note, the method of VAP-IP abstraction for the SDD review [6] was somewhat unique in that these authors had contacted investigators of the original SDD studies to obtain 'intention to treat' data. Hence, the SDD data includes missing data for 25 of the 36 SDD studies with published data used for the remaining 11 studies. However, applying the benchmark 95% prediction range to the VAP-IP data as published in all 33 studies yields similar discrepancies [12].

The third analytic issue is that the VAP-IP is proportion data arising from groups with varying denominators. Transformation to logits and weighting by the inverse variance as a method of adjusting for variable study size are standard methods for analysis of proportion data [15,16].

The fourth issue is that the studies vary considerably in the intervention under study. It should be noted that profiling the component groups of the prevention studies against the benchmark is the objective of the analysis here rather than estimating the summary effect size for the interventions under study. In this regard, the control groups are of particular interest. If there is no contextual effect associated with the study of SDD within an ICU, it would be expected that the control groups of concurrent design SDD studies would have VAP-IP's similar not only to each other, but to the benchmark and also to the VAP-IP's of control groups of studies of other prevention methods.

The fifth issue is that the quality scores of the studies as rated in each systematic review varied. Also, different scales of study quality were used in each of the systematic reviews. As a consequence, a majority quality score as rated by each systematic review was used as a unified rating of highest study quality. Paradoxically, the disparities in VAP-IP noted here are most apparent in comparisons limited to the highest quality studies.

The sixth issue is the heterogeneity (over-dispersion) in event rates arising from different patient populations in different centres. This is apparent in all of the summary ranges here in that all have I^2 ^values above 75% which indicate high levels of heterogeneity [132]. Heterogeneity has been a major obstacle in the context of profiling the performance of hospitals and surgeons toward the identification of individual outlier performers. Adjusting for patient risk is an important consideration in profiling, but this is problematic when comparing multiple centres [133]. It should be noted that identification of individual outlier performers is not an objective of this analysis but rather the estimation of the overall VAP-IP range among the component groups that comprise an entire evidence base and the identification of group level explanatory variables in the meta-regression models of VAP-IP.

A more recent development in relation to managing heterogeneity is to measure it using random effects methods [13,14,132]. With random effects methods, both the variance arising from between groups (heterogeneity, tau^2^) versus that from within groups (sampling, SE) are estimated and both types of variability are incorporated in the calculation of the 95% prediction intervals with as a result, more conservative (wider) prediction intervals than would be derived using traditional fixed effects methods which do not take heterogeneity into account.

There were 19 different topical SDD intervention regimens studied. The most common regimen used in the studies included here was a topical combination of polymyxin, tobramycin and amphotericin together with, for 13 of the SDD intervention groups, a parenteral antibiotic [134]. Given the heterogeneity in the SDD treatments, surprisingly the IQR (Table 1) was wider and the tau^2 ^(Table 3) was higher for the control groups of the SDD studies than for the corresponding intervention groups and also versus the control and intervention groups of studies of three different types of non-antibiotic prevention methods. These are paradoxical findings.

The seventh issue is observer bias and the lack of an objective gold standard for VAP. In part this issue relates to study design and the blinding of observers and adequate concealment of group allocation, factors that have been assessed as part of the study quality ratings used in each of the systematic reviews. More particularly, lack of an objective gold standard is an important issue in the case of VAP for which there are several definitions in use, with those that require bronchoscopic based sampling being possibly a more specific but less sensitive diagnostic standard [135].

Additional to this is that some of the studies included patients who were not ventilated, which is problematic for the diagnosis of VAP. Also, the proportion of patients admitted for trauma, used here as a surrogate for the patient mix, ranged between 0% and 100% among the studies. These issues in VAP diagnosis have been identified as group level factors in meta-regression models 2, 3, 5 and 6.

### Limitations of this analysis

There are several limitations of this analysis. The random effects method of analysis presumes that the groups in each summation are representative of a 'random' selection of an undefinable super-population of groups. The VAP-IP benchmark derived here may only be representative of patient groups as found within systematic reviews.

Only nine reviews were used in this analysis and other smaller reviews have not been included. However, other reviews applicable to this patient group can be tested against the 95% prediction range derived from the benchmark groups here. For example, systematic reviews of kinetic bed [136] therapy and topical chlohexidene [137] as methods for the prevention of VAP had identified 10 and 7 studies respectively. Of the 17 studies identified in these two systematic reviews, only two studies, one from each systematic review, had a control group with a VAP-IP above 47.3%, the upper 95% limit of the prediction range derived from the benchmark groups here, whereas three had an intervention group VAP-IP below the lower 95% limit of the prediction range derived from the benchmark groups.

Also, studies of SDD which had a non-concurrent design have not been included in the meta-regression. This would help to test the postulated contextual effect of SDD. Among the SDD studies included here, three have a third non-concurrent control group arm in addition to the two concurrent arms [101,104,131]. The VAP-IP of all three of these non-concurrent control groups is less than 24% [12].

A further limitation was that the number of group level factors that could be explored was limited by those that were readily available and identified for all the groups in the analysis. Origin from a European country and year of publication were tested and found not to be significant in preliminary analyses (data not shown). Other factors such as the prevalence of antibiotic use have not been explored beyond that accounted for by duplex study design. Also the duration of mechanical ventilation has not been considered beyond the group average, which appeared to be similar across the strata (Table 1). The appropriate investigation of these factors would require patient level data to control for the possible influence of ecological bias [138].

There are three possible interpretations of these paradoxical findings. First, publication and citation bias need to be considered. Deriving the benchmark from four non-systematic and one systematic reviews was done to test the stability of the benchmark estimate (Table 2). In replications of the benchmark range from these five reviews separately this varies by no more than five percentage points.

Given that 11 of the 33 control groups from studies of SDD are above the upper limit of the 95% prediction range of the benchmark where only 2.5% of the distribution would be predicted to be found, this could be taken to indicate a deficit of 407 groups below the upper limit of the 95% prediction range of the benchmark {407 = (11 * 97.5/2.5)- 22)}. This estimate corresponds to an earlier test for publication bias using a funnel plot method which indicated a deficit of >500 'inlier' groups with VAP-IP < 45% from studies of SDD that had been unpublished or were otherwise 'missing' [12].

A second interpretation is that the possible impacts of unmeasured and unknown patient level risk factors for VAP-IP have not been evaluated in this analysis. However, for such a risk factor to account for the discrepancies between the VAP-IP of the control groups of SDD studies versus the benchmark groups is unlikely. Such putative risk factors would need to be consistently strong across the range of studies and yet have a profoundly uneven distribution between the SDD studies versus other studies. This is in contrast to the inconsistent strength and direction of the known VAP risk factors [2,3].

For example, duration of mechanical ventilation is the strongest patient level risk factor for VAP with increases of approximately 2 per 100 patients per day of ventilation during the second week of ventilation [139]. The discrepancies in VAP-IP noted here between the control groups of SDD studies versus the benchmark would equate to a difference in mean duration of ventilation across all groups of 6.8 days.

A third interpretation is a possible contextual effect of SDD. The possibility of contextual effects due to cross colonization and infection within the ICU environment resulting from SDD use as was postulated in the original study of SDD [10] needs to be considered [140]. SDD is known to alter the colonization among recipients [141,142]. However, identifying cross colonization within a single study is difficult. A major limitation toward testing this postulate is that colonization pressure [143] and cross colonization, two crucial intermediary steps, have not been measured in any of these studies.

## Conclusions

The VAP-IP among control groups of SDD studies is more variable and the mean is >50% greater than other groups within the evidence base including the VAP benchmark. These paradoxical findings cannot be accounted for through group level adjustments for proportion of trauma admissions, mode of VAP diagnosis and proportion of patients receiving prolonged ventilation.

Apart from major publication bias, or the effect of a major and as yet unidentified and mal-distributed patient level VAP risk factor, or the effect of in-apparent outbreaks [140], these paradoxical discrepancies cannot be explained. The interpretation of the studies of SDD treatments cannot proceed without further consideration that SDD may have a contextual effect as originally postulated [10].

## Key messages

• A VAP-IP benchmark derived from 45 observational (benchmark) groups of mechanically ventilated patients is 22.1%.

• The mean VAP-IP of 35 control groups from studies of three non-antimicrobial methods of VAP prevention versus 33 control groups of studies of SDD are, respectively, within 2 percentage points of versus more than 13 percentage points higher than the benchmark.

• By contrast, the mean VAP-IP of 35 intervention groups studies of non-antimicrobial methods versus 34 SDD intervention groups are each within six percentage points of the benchmark.

• The paradoxical findings are most apparent in comparisons limited to the highest quality studies.

• These observations cannot readily be accounted for with adjustments for group level factors such as proportion of trauma admissions, mode of diagnosis and study quality.

## Abbreviations

CI: confidence interval; ICU: Intensive Care Unit; IQR: interquartile range; MV: mechanical ventilation; SDD: selective digestive decontamination; SE: standard error; VAP: Ventilator associated pneumonia; VAP-IP: VAP-Incidence proportion.

## Competing interests

The author declares that he has no competing interests.

## Authors' contributions

JH produced the design of the study, performed the statistical analysis, wrote the manuscript and read and approved the final manuscript.

## Supplementary Material

Additional file 1 **VAP-IP data for benchmark groups**. Click here for file

Additional file 2 **VAP-IP data for component groups of studies of non-antibiotic methods of VAP prevention**. Click here for file

Additional file 3 **VAP-IP data for component groups of studies of SDD**. Click here for file
