# Supplementary material for: Paradoxical ventilator associated pneumonia incidences among selective digestive decontamination studies versus other studies of mechanically ventilated patients: benchmarking the evidence base
Source: Crit Care. 2011 Jan 7;15(1):R7. doi: 10.1186/cc9406 (PMC3222036; doi:10.1186/cc9406)
Supplement: Additional file 1 — VAP-IP data for benchmark groups. [file cc9406-S1.DOC]

**Table S1: Benchmark groups ^a^**

| Study | Group size | VAP-IP (%) | Citation source of VAP-IP data |
| --- | --- | --- | --- |
| Akca et al. [19] | 260 | 31 | Safdar et al. [5] |
| Apostolopoulou et al. [20] **^b^** | 175 | 32 | Safdar et al. [5] |
| Baker et al. [21] | 514 | 6 | Chastre et al. [3] |
| Baraibar et al. [22] **^b^** | 707 | 21 | Cook et al. [2] |
| Beck-Sague et al. [23] | 145 | 10 | Safdar et al. [5] |
| Bercault et al. [24] **^b^** | 1144 | 12 | Safdar et al. [5] |
| Bochicchio et al. [25] | 678 | 21 | Safdar et al. [5] |
| Bonten et al. [26] **^b^** | 64 | 17 | Bergmans et al. [4] |
| Braun et al. [27] | 66 | 23 | George et al. [1] |
| Craven et al. [28] | 233 | 21 | George et al. [1] |
| Daschner et al. [29] **^b^** | 84 | 43 | George et al. [1] |
| Daumal et al. [30] **^b^** | 361 | 15 | Safdar et al. [5] |
| de Latore et al. [31] **^b^** | 80 | 15 | Bergmans et al. [4] |
| Eggimann et al. [32] **^b^** | 452 | 23 | Safdar et al. [5] |
| Elatrous et al. [33] **^b^** | 73 | 38 | Cook et al. [2] |
| Fagon'89 et al. [34] **^b^** | 567 | 9 | Chastre et al. [3] |
| Fagon'96 et al. [35] **^b^** | 1978 | 17 | Chastre et al. [3] |
| Garrouste-Orgas et al.[36] **^b^** | 86 | 36 | Bergmans et al. [4] |
| Ibrahim'00 et al. [37] | 1882 | 21 | Safdar et al. [5] |
| Ibrahim'01 et al. [38] | 880 | 15 | Safdar et al. [5] |
| Jacobs et al. [39] **^b^** | 24 | 54 | George et al. [1] |
| Jimenez et al. [40] **^b^** | 77 | 23 | George et al. [1] |
|  |  |  |  |

**Table S1 (Continued): Observational** study groups

| Study | Group size | VAP-IP (%) | Citation source of VAP-IP data† |
| --- | --- | --- | --- |
| Kanafani et al. [41] | 70 | 47 | Safdar et al. [5] |
| Kappstein et al. [42] **^b^** | 270 | 29 | Safdar et al. [5] |
| Kingston et al. [43] | 24 | 42 | Bergmans et al. [4] |
| Kollef’93 et al. [44] | 277 | 16 | Cook et al. [2] |
| Kollef’95 et al. [45] | 314 | 28 | Safdar et al. [5] |
| Kollef’97 et al. [46] | 521 | 15 | Bergmans et al. [4] |
| Langer et al. [47] **^b^** | 724 | 23 | Cook et al. [2] |
| Mauritz et al. [48] **^b^** | 34 | 21 | George et al. [1] |
| Memish et al. [49] | 202 | 25 | Safdar et al. [5] |
| Moine et al. [50] **^b^** | 764 | 12 | Safdar et al. [5] |
| Papazain et al. [51] **^b^** | 586 | 17 | Bergmans et al. [4] |
| Rashkin et al. [52] | 61 | 11 | George et al. [1] |
| Rello’91 et al. [53] **^b^** | 264 | 22 | Safdar et al. [5] |
| Rello’92 et al. [54] **^b^** | 161 | 26 | Bergmans et al. [4] |
| Rello’94 et al. [55] **^b^** | 568 | 13 | Cook et al. [2] |
| Rello’96 et al. [56] **^b^** | 83 | 25 | Cook et al. [2] |
| Rodriguez et al. [57] | 294 | 44 | Safdar et al. [5] |
| Ruiz-Santana et al. [58] **^b^** | 1005 | 31 | George et al. [1] |
| Salata et al. [59] | 51 | 41 | Chastre et al. [3] |
| Sofianou et al. [60] **^b^** | 198 | 34 | Safdar et al. [5] |
| Tejada-Artigas et al. [61] **^b^** | 103 | 22 | Chastre et al. [3] |
| Timsit et al. [62] **^b^** | 387 | 14 | Chastre et al. [3] |
| Torres et al. [63] **^b^** | 322 | 24 | George et al. [1] |

VAP-IP, Ventilator associated pneumonia incidence proportion.

Footnotes

1. The following systematic reviews were the source for these studies and VAP-IP data; George, 1993 [1] (Table 1), Cook and Kollef, 1998 [2] (Table 1), Chastre and Fagon, [3] 2002 (Table 1), Bergmans and Bonten, [4] 2004 (Table 22.5), Safdar et al., [5] 2005 (Table 1).
2. Originating from a member state of the European Union as at 2010 or Switzerland or Norway.
