# Supplementary material for: Paradoxical ventilator associated pneumonia incidences among selective digestive decontamination studies versus other studies of mechanically ventilated patients: benchmarking the evidence base
Source: Crit Care. 2011 Jan 7;15(1):R7. doi: 10.1186/cc9406 (PMC3222036; doi:10.1186/cc9406)
Supplement: Additional file 2 — VAP-IP data for component groups of studies of non-antibiotic methods of VAP prevention. [file cc9406-S2.DOC]

**Table S2: Studies of non-antimicrobial-based methods of VAP prevention ^a^**

| Source **^b, c^** | Control groups | | |  | Intervention groups | | |  |
| --- | --- | --- | --- | --- | --- | --- | --- | --- |
|  | Treatment **^d^** | Group size | VAP-IP  (%) |  | Treatment **^d^** | Group size | VAP-IP  (%) |
| Adams et al. [64] **^c^** | OTSS | 10 | 0 |  | CTSS | 10 | 0 |  |
| Ben-Menachem et al. [65] **^b^** | No sucralfate | 100 | 6 |  | No ranitidine | 100 | 12 |  |
| Boots '97 et al. [66] | HH | 41 | 17 |  | HME | 75 | 19 |  |
| Boots'06 et al. [67] | HH | 191 | 12 |  | HME | 190 | 13 |  |
| Branson et al. [68] **^b^** | HH | 54 | 6 |  | HME | 49 | 6 |  |
| Combes et al. [69] **^c^** | OTSS | 50 | 18 |  | CTSS | 54 | 7 |  |
| Conrad et al. [70] | OTSS | 17 | 35 |  | CTSS | 16 | 38 |  |
| Cook et al. [71] **^b^** | No sucralfate | 596 | 19 |  | No ranitidine | 604 | 16 |  |
| Deppe et al. [72] **^b^** | OTSS | 38 | 29 |  | CTSS | 46 | 26 |  |
| Dreyfuss et al. [73] **^c^** | HH | 70 | 11 |  | HME | 61 | 10 |  |
| Eddleston '91 et al. [74] **^b, c^** | No sucralfate | 30 | 33 |  | No ranitidine | 30 | 10 |  |
| Eddleston '94 et al. [75] **^c^** | No sucralfate | 12 | 0 |  | No ranitidine | 14 | 7 |  |
| Hanisch et al. [76] **^b, c^** | No sucralfate | 57 | 18 |  | No ranitidine | 57 | 21 |  |
| Hurni et al. [77] **^c^** | HH | 56 | 13 |  | HME | 59 | 9 |  |
| Johnson et al. [78] | OTSS | 19 | 53 |  | CTSS | 16 | 50 |  |
| Kirton et al. [79] **^b^** | HH | 140 | 16 |  | HME | 140 | 6 |  |
| Kollef'98 et al. [80] **^b^** | HH | 147 | 10 |  | CTSS | 163 | 9 |  |
| Lacherade et al. [81] **^b^** | HH | 184 | 29 |  | HME | 185 | 25 |  |
|  |  |  |  |  |  |  |  |  |

**Table S2 (continued): Studies of non-antimicrobial-based methods of VAP prevention**

| Source | Control groups | | |  | Intervention groups | | |  |
| --- | --- | --- | --- | --- | --- | --- | --- | --- |
|  | Treatment **^b^** | Group size | VAP-IP  (%) |  | Treatment **^b^** | Group size | VAP-IP  (%) |
| Laggner et al. [82] | No sucralfate | 16 | 13 |  | No ranitidine | 16 | 0 |  |
| Lorente'05 et al. [83] **^b, c^** | OTSS | 233 | 18 |  | CTSS | 210 | 21 |  |
| Lorente'06 et al. [84] | OTSS | 221 | 14 |  | CTSS | 236 | 14 |  |
| Lorente'06 et al. [85] **^b, c^** | HH | 51 | 16 |  | HME | 53 | 40 |  |
| Martin et al. [86] **^c^** | HH | 42 | 19 |  | HME | 31 | 7 |  |
| Memish et al. [87] | HH | 120 | 16 |  | HME | 123 | 11 |  |
| Metz et al. [88] **^b^** | No sucralfate | 84 | 14 |  | No ranitidine | 79 | 19 |  |
| Mustafa et al. [89] **^c^** | No sucralfate | 16 | 56 |  | No ranitidine | 15 | 20 |  |
| O'Keefe et al. [90] **^b^** | No sucralfate | 49 | 29 |  | No ranitidine | 47 | 21 |  |
| Pickworth et al. [91] **^b^** | No sucralfate | 44 | 11 |  | No ranitidine | 39 | 15 |  |
| Prod'hom_R et al. [92] **^b, c^** | No sucralfate | 80 | 26 |  | No ranitidine | 83 | 12 |  |
| Rabitsch et al. [93] **^b, c^** | OTSS | 12 | 42 |  | CTSS | 12 | 0 |  |
| Roustan et al. [94] **^c^** | HH | 61 | 15 |  | HME | 55 | 9 |  |
| Thomasen R et al. [95] | No sucralfate | 80 | 34 |  | No ranitidine | 80 | 38 |  |
| Topeli et al. [96] **^c^** | OTSS | 37 | 24 |  | CTSS | 41 | 32 |  |
| Welte et al. [97] **^c^** | OTSS | 25 | 64 |  | CTSS | 27 | 33 |  |
| Zeitoun et al. [98] | OTSS | 24 | 46 |  | CTSS | 23 | 30 |  |

VAP-IP, Ventilator associated pneumonia incidence proportion; OTSS, open tracheal suction system; CTSS, closed tracheal suction system; HH, Heated humidifier; HME, heat and moisture exchanger

Footnotes

1. The following systematic reviews were the source for these studies and VAP-IP data; Messori et al., [7] 2000 (Tables 5, 6 & 7), Subirana et al., [8] 2007 (Table 7), and Siempos et al., [9] 2007 (Table 2).
2. Studies that received a majority quality score in the source systematic review.
3. Originating from a member state of the European Union as at 2010 or Switzerland or Norway
4. Treatment abbreviations; OTSS, open tracheal suction system; CTSS, closed tracheal suction system; HH, Heated humidifier; HME, heat and moisture exchanger
