# Supplementary material for: Paradoxical ventilator associated pneumonia incidences among selective digestive decontamination studies versus other studies of mechanically ventilated patients: benchmarking the evidence base
Source: Crit Care. 2011 Jan 7;15(1):R7. doi: 10.1186/cc9406 (PMC3222036; doi:10.1186/cc9406)
Supplement: Additional file 3 — VAP-IP data for component groups of studies of SDD. [file cc9406-S3.DOC]

**Table S3: Studies of SDD studies as a method of VAP prevention ^a^**

| Source | Control groups | | |  | Intervention groups | | |  |
| --- | --- | --- | --- | --- | --- | --- | --- | --- |
|  | Treatment **^b^** | Group size | VAP-IP  (%) |  | Treatment **^b^** | Group size | VAP-IP  (%) |
| Abele-Horn et al. [99] ^c^ |  | 30 | 77 |  | PTA-Ctx | 58 | 22 |  |
| Aerdts et al. [100] ^c^ |  | 60 | 48 |  | PNoA-Ctx | 28 | 4 |  |
| Bergmans et al. [101] ^c^ | Pl | 139 | 27 |  | PGV | 87 | 10 |  |
| Blair et al. [102] ^c^ |  | 170 | 22 |  | PTA-Ctx | 161 | 7 |  |
| Boland et al. [103] ^c^ | Pl | 32 | 53 |  | PTNy-Ctx | 32 | 44 |  |
| Brun_Buisson et al. [104] ^c^ |  | 68 | 9 |  | PNeNa | 65 | 5 |  |
| Camus et al. [105] ^c^ | Pl | 126 | 42 |  | PT | 130 | 41 |  |
| Cockerill et al. [106] |  | 75 | 16 |  | PGNy-Ctx | 75 | 5 |  |
| Ferrer et al. [107] ^c, d^ | Pl-Ctx | 50 | 22 |  | PTA-Ctx | 51 | 14 |  |
| Finch et al. [108] ^c^ |  | 24 | 29 |  | PGA-Ctx | 20 | 20 |  |
| Gastinne et al. [109] ^c^ | Pl | 225 | 15 |  | PTA | 220 | 12 |  |
| Georges et al. [110] ^c^ | Pl | 33 | 45 |  | PNeA | 31 | 13 |  |
| Hammond et al. [111] ^d^ | Pl-Ctx | 160 | 19 |  | PTA-Ctx | 162 | 15 |  |
| Jacobs et al. [112] ^c^ |  | 46 | 9 |  | PTA-Ctx | 45 | 0 |  |
| Kerver et al. [113] ^c^ |  | 47 | 66 |  | PTA-Ctx | 49 | 10 |  |
| Korinek et al. [114] ^c^ | Pl | 95 | 39 |  | PTA-V | 96 | 21 |  |
| Krueger et al. [115] ^c^, **^e^** | Pl | 262 | 57 |  | PG-Cip | 265 | 34 |  |
| Laggner et al. [116] ^c^ | Pl | 34 | 12 |  | GA | 33 | 3 |  |
| Lingnau et al. [117] ^c, d^ | Pl-Cip | 177 | 40 |  | PCipA-Cip | 90 | 38 |  |
| Lingnau et al. [117] ^c, d^ |  |  |  |  | PTA-Cip | 90 | 42 |  |

**Table S3 (continued): Studies of SDD studies as a method of VAP prevention**

| Source | Control groups | | |  | Intervention groups | | |  |
| --- | --- | --- | --- | --- | --- | --- | --- | --- |
|  | Treatment **^b^** | Group size | VAP-IP  (%) |  | Treatment **^b^** | Group size | VAP-IP  (%) |
| Palomar et al. [118] ^c^ |  | 49 | 51 |  | PTA-Ctx | 50 | 20 |  |
| Pneumatikos et al. [119] ^c^ | Pl | 30 | 53 |  | PTA | 31 | 16 |  |
| Pugin et al. [120] ^c^**^, e^** | Pl | 41 | 59 |  | PNeV | 38 | 11 |  |
| Quinio et al. [121] ^c^ | Pl | 73 | 52 |  | PGA | 76 | 25 |  |
| Rocha et al. [122] ^c^ | Pl | 54 | 46 |  | PTA-Ctx | 47 | 15 |  |
| Rodriguez-Roldan et al. [123] ^c^ | Pl | 17 | 65 |  | PTNeA | 14 | 7 |  |
| Sanchez-Garcia et al. [124] ^c^, **^d, e^** | Pl | 140 | 43 |  | PGA-Ctx | 131 | 24 |  |
| Stoutenbeek’96 et al. [125] ^c, d^, **^e^** | Pl-Ctx | 42 | 19 |  | PTA-Ctx | 49 | 4 |  |
| Stoutenbeek’07 et al. [126] ^c^ |  | 200 | 50 |  | PTA-Ctx | 201 | 31 |  |
| Ulrich et al. [127] ^c^ | Pl | 57 | 46 |  | PNoA-Tr | 55 | 13 |  |
| Unertl et al. [128] ^c^ |  | 20 | 45 |  | PGA | 19 | 5 |  |
| Verwaest et al. [129] ^c^ |  | 185 | 22 |  | OA-O | 193 | 11 |  |
| Weiner et al. [130] | Pl | 31 | 26 |  | PGNy | 30 | 27 |  |
| Winter et al. [131] ^c^ |  | 92 | 18 |  | PTA-Cz | 91 | 3 |  |
|  |  |  |  |  |  |  |  |  |

VAP-IP, Ventilator associated pneumonia incidence proportion; Pl, topical placebo; Pl-Ctx, topical placebo and systemic cefotaxime; Pl-Cip, topical placebo and systemic ciprofloxacin; PTA-Ctx, topical polymyxin, tobramycin and amphotericin with systemic cefotaxime; PNoA-Ctx, topical polymyxin, norlfoxacin and amphotericin with systemic cefotaxime; PGV, topical polymyxin, gentamicin and vancomycin; PTNy-Ctx; topical polymyxin, tobramycin and nystatin with systemic cefotaxime; PNeNa, topical polymyxin neomycin and nalidixic acid; PT, topical polymyxin, and tobramycin; PGNy-Ctx, topical polymyxin, gentamicin, nystatin and systemic cefotaxime; PTA, topical polymyxin, tobramycin and amphotericin; PNeA, topical polymyxin, neomycin and amphotericin; PTA-V, topical polymyxin, tobramycin and amphotericin and systemic vancomycin; PG-Cip, topical polymyxin, gentamicin, and systemic ciprofloxacin; GA, topical gentamicin and amphotericin; PCipA-Cip, topical ciprofloxacin, amphotericin and systemic ciprofloxacin; PTA-Cip, topical polymyxin, tobramycin and amphotericin and systemic ciprofloxacin; PneV, topical polymyxin neomycin and vancomycin; PGA, topical polymyxin, gentamicin, and amphotericin; PTNeA, topical polymyxin, tobramycin, neomycin and amphotericin; PNoA-Tr, topical polymyxin, norlfoxacin amphotericin and systemic trimethoprim; OA-O, topical oflaxcin, amphotericin and systemic oflaxcin; PGNy, topical polymyxin, gentamicin, and amphotericin; PTA-Cz, topical polymyxin, tobramycin and amphotericin with systemic ceftazidime.

Footnotes

1. The following systematic review was the source for these studies and VAP-IP data; Liberati et al., [6] 2009 (Analysis 1.5, & 2.5)
2. Treatment abbreviations; Pl, topical placebo; Pl-Ctx, topical placebo and systemic cefotaxime; Pl-Cip, topical placebo and systemic ciprofloxacin; PTA-Ctx, topical polymyxin, tobramycin and amphotericin with systemic cefotaxime; PNoA-Ctx, topical polymyxin, norlfoxacin and amphotericin with systemic cefotaxime; PGV, topical polymyxin, gentamicin and vancomycin; PTNy-Ctx; topical polymyxin, tobramycin and nystatin with systemic cefotaxime; PNeNa, topical polymyxin neomycin and nalidixic acid; PT, topical polymyxin, and tobramycin; PGNy-Ctx, topical polymyxin, gentamicin, nystatin and systemic cefotaxime; PTA, topical polymyxin, tobramycin and amphotericin; PNeA, topical polymyxin, neomycin and amphotericin; PTA-V, topical polymyxin, tobramycin and amphotericin and systemic vancomycin; PG-Cip, topical polymyxin, gentamicin, and systemic ciprofloxacin; GA, topical gentamicin and amphotericin; PCipA-Cip, topical ciprofloxacin, amphotericin and systemic ciprofloxacin; PTA-Cip, topical polymyxin, tobramycin and amphotericin and systemic ciprofloxacin; PneV, topical polymyxin neomycin and vancomycin; PGA, topical polymyxin, gentamicin, and amphotericin; PTNeA, topical polymyxin, tobramycin, neomycin and amphotericin; PNoA-Tr, topical polymyxin, norlfoxacin amphotericin and systemic trimethoprim; OA-O, topical oflaxcin, amphotericin and systemic oflaxcin; PGNy, topical polymyxin, gentamicin, and amphotericin; PTA-Cz, topical polymyxin, tobramycin and amphotericin with systemic ceftazidime.
3. Originating from a member state of the European Union as at 2010 or Switzerland or Norway
4. Duplex study, i.e. systemic antimicrobial routinely administered to control group patients.
5. Studies that received a majority quality score in the source systematic review.
